# Safety and Effectiveness of Total Thyroidectomy and Its Comparison with Subtotal Thyroidectomy and Other Thyroid Surgeries: A Systematic Review

**DOI:** 10.1155/2016/7594615

**Published:** 2016-02-24

**Authors:** Ashwini Aithal Padur, Naveen Kumar, Anitha Guru, Satheesha Nayak Badagabettu, Swamy Ravindra Shanthakumar, Murlimanju Bukkambudhi Virupakshamurthy, Jyothsna Patil

**Affiliations:** ^1^Department of Anatomy, Melaka Manipal Medical College, Manipal University, Manipal Campus, Manipal 576104, India; ^2^Department of Anatomy, Kasturba Medical College, Mangalore, Manipal University, Manipal 575001, India

## Abstract

Diseases associated with the thyroid gland are one of the most frequently seen endocrine disorders across the globe. Total thyroidectomy is currently the preferred treatment for many thyroid diseases. Controversies exist among surgeons regarding safety of total thyroidectomy due to the risk associated with it like postoperative hypoparathyroidism or recurrent laryngeal nerve damage. Since, in the recent years, the incidence of thyroidectomy is in increasing trend in south Indian population, this review aims to study the available data regarding the appropriateness and safety of total thyroidectomy and compares it with subtotal thyroidectomy and other thyroid surgeries. This is a retrospective comprehensive review of various articles and publications regarding total and partial thyroidectomy performed across the world. Many retrospective studies and few prospective studies suggest that the incidence of transient hypocalcemia is higher after total thyroidectomy than after subtotal thyroidectomy, but the incidence of other complications including recurrent laryngeal nerve palsy and postoperative hematoma is not significantly different between the two procedures. Hence in our review we found that total thyroidectomy is safe and cost effective with low complication rates and provides little significant advantage of being safer procedure compared to subtotal thyroidectomy.

## 1. Introduction

The incidence of thyroid disease in general population is enormous. Thyroid disorders are the most frequently encountered endocrine diseases in India [1]. The thyroid is a small endocrine gland which produces hormones that regulate various metabolic activities of the body [2]. Thyroid diseases can be grouped into benign and malignant types. In benign cases, the common diseases encountered are thyroiditis (mostly Hashimoto thyroiditis), goiter, thyroid adenoma, and so forth. Total thyroidectomy is a surgical procedure which is performed to treat various thyroid diseases wherein the thyroid gland is removed. But the use of total thyroidectomy procedure is considered not to be safe for thyroid carcinomas and also for treatment of few benign diseases because of the risks involved [3]. If thyroidectomy is undertaken, care must be taken when ligating the superior and inferior thyroid artery to avoid damage to adjacent nerves. Ligation of the superior thyroid artery close to the superior pole of thyroid gland is considered safe. Safest approach is to identify the branches of superior thyroid artery and avoid ligating the main trunk as in majority of cases superior laryngeal nerve lies close to the main trunk. The external laryngeal nerves run close to superior thyroid artery and recurrent laryngeal nerve runs close to inferior thyroid artery [1]. Surgeons avoid this procedure due to the possible complications associated with it such as permanent recurrent laryngeal nerve palsy or permanent hypoparathyroidism. So, partial or subtotal thyroidectomy is another procedure which is preferred and currently being performed for benign thyroid diseases [4]. In recent years the use of nerve monitors and stimulators has been advocated but their usefulness still remains highly uncertain. In fact one study reports that the surgeons were able to use it only to identify superior laryngeal nerve and it did not aid them in the anatomical dissection of recurrent laryngeal nerve [5]. Few minimally invasive methods are now widely used for performing endocrine surgeries.

Total thyroidectomy provides the advantages of eliminating the risk of recurrence and hence an increasing number of total thyroidectomies are currently being performed for benign cases. Knowledge about the clinical profiles of thyroidectomy cases and understanding the postthyroidectomy complications are an important milestone in public health. Since, in the recent years, the incidence of thyroidectomy is in increasing trend in south Indian population, our aim was to assess the safety of total thyroidectomy. Safety of thyroidectomy procedures is a major concern for both patients and physicians. Thus, this review aims to study the available data regarding the appropriateness and safety of total thyroidectomy and compares it with subtotal thyroidectomy and other thyroid surgeries.

## 2. Materials and Methods

This review was prepared by downloading various articles regarding total and partial thyroidectomy from several search engines like PubMed, Medline, Scopus, Google Scholar, and so forth. The search was limited to clinical trials, controlled clinical trials, comparative studies, and randomized controlled trials. Only articles pertaining to and relevant to the effectiveness and complications encountered during thyroidectomy were included in this review. Articles which compared total thyroidectomy with subtotal thyroidectomy and near total thyroidectomy on various outcomes were also reviewed. Primary outcomes were defined as prevalence of recurrence of thyroid diseases after thyroidectomy. Secondary outcomes were defined as incidence of complications or morbidity following thyroidectomy. Factors such as safety, outcomes of the surgery, and postoperative complications related to thyroidectomy were evaluated. A total of 41 studies were reviewed.

## 3. Results

**Figure 1:**
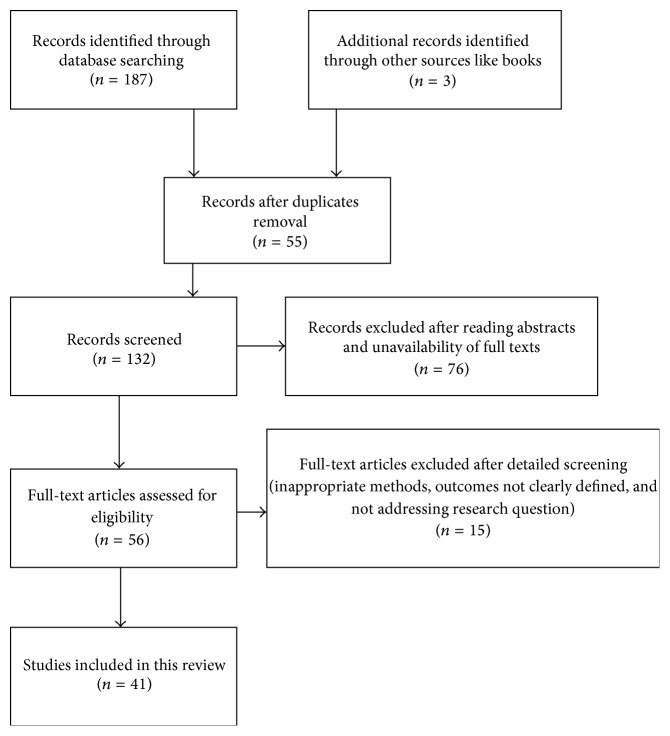
Flow diagram of literature search.

Records identified through database searching were 187. These records were screened and 56 full-text articles were assessed for eligibility. After detailed screening 41 articles were included in this review (Figure 1). Most articles which were reviewed were retrospective studies or nonrandomized controlled trials. Few articles reported that requirement of reoperation was significantly less often in total thyroidectomy when compared to subtotal thyroidectomy or hemitotal thyroidectomy. Studies have reported complications such as hypoparathyroidism or hypocalcemia and recurrent laryngeal nerve injury following thyroidectomy. Gough and Wilkinson have reported that recurrent laryngeal nerve palsy and permanent hypoparathyroidism are the most common complications following total thyroidectomy surgery which account for 0.7% and 2.2%, respectively [6]. In a review done by Jessie and Harrison, they have found that reported rate of transient hypoparathyroidism ranged from 5 to 71% while the rate of permanent hypoparathyroidism ranged from 0 to 3.5% [7]. Perzik in his work reported an incidence of nerve injury which is only 0.4% with no hypoparathyroidism [8]. We found eight studies which compared the incidences of recurrent laryngeal nerve injury in total thyroidectomy, subtotal thyroidectomy, near total thyroidectomy, and bilateral subtotal thyroidectomy. They indicate that the complication was higher when performing total thyroidectomy. These results are shown in Table 1. Few studies which compare total thyroidectomy with subtotal thyroidectomy mostly provide level IV and some level II evidence that subtotal thyroidectomy is a safer procedure in terms of lesser rates of transient hypocalcemia, although rates of permanent complications do not differ in both. We observed that there are few studies which report high complication rates following thyroidectomy while few studies report very low complication rates. In addition, there are studies which report that there was no significant difference in complication rates among patients who underwent total thyroidectomy when compared with those patients undergoing subtotal thyroidectomy [8–10]. Nevertheless, the complication rates were observed to be higher if the surgery was performed by surgeons who are not specialists in endocrine surgery [6]. However, repeat surgery for recurrent thyroid disease was seen to carry significantly higher risks when compared to the initial surgery. The incidences of recurrent laryngeal nerve palsy and permanent hypoparathyroidism in such cases are found to be as high as 20.0% and 3.4% [11, 12]. The incidence of permanent recurrent laryngeal nerve palsy in 4 clinical trials was lower for subtotal thyroidectomy compared with total thyroidectomy. Permanent recurrent laryngeal nerve palsy in these studies occurred in 0.8% of subtotal thyroidectomy patients compared to 0.7% of total thyroidectomy patients (*p* = 0.69; *n* = 1275). A review article comparing total thyroidectomy with subtotal thyroidectomy for multinodular goiter indicated that goiter recurrence was lower in the total thyroidectomy group (0.2%) compared to subtotal thyroidectomy group (8.4%) with *p* < 0.000 [13]. To summarize, many retrospective studies providing level IV evidence and few prospective studies providing level III evidences suggest that the incidence of transient hypocalcemia is higher after total thyroidectomy than after subtotal thyroidectomy, but the incidence of other complications including recurrent laryngeal nerve palsy and postoperative hematoma is not significantly different between the two procedures. Although contrary views exist, most of articles have stressed the fact that total thyroidectomy is safe and had fewer complications when compared to subtotal or partial thyroidectomy.

## 4. Discussion

Diseases of thyroid gland are of great importance because they are a challenge for medical or surgical management. Total thyroidectomy is considered as the usual surgical procedure to treat thyroid diseases. The principal diseases of the thyroid gland are goitre, hypothyroidism, hyperthyroidism, thyroiditis, and neoplasms [14].

Reports indicate that the incidence of benign and malignant lesions in surgically treated thyroid swellings depends on the person's lifestyle and varies widely from one geographical area to another [15]. The prevalence of thyroid diseases is found to increase linearly as age advances and most of the thyroid diseases are found to be more common in women [16]. In a retrospective analysis done by Rumstadt et al., a total of 253 cases which presented with goitre were reported, of which 134 hemithyroidectomies, 108 hemithyroidectomies combined with subtotal contralateral resection, and 11 total thyroidectomies were performed [17]. This shows that thyroid diseases can be treated surgically using procedures other than total thyroidectomy.

Thyroidectomy surgery is said to be the most common cause of bilateral vocal cord paralyses. A study suggests that thyroidectomy surgery is one of the main factors which cause bilateral vocal cord injury [18]. There is always a higher risk of damaging the recurrent laryngeal nerve during a second surgery due to the presence of the scar tissue which would be left behind after the first surgery and also due to few degenerative changes associated with the scar tissue [19]. There are reports of high rates of temporary (15.5% to 23.6%) and permanent (2.6% to 15.5%) damage to the nerve following secondary thyroidectomy [20]. Prior knowledge, accurate exposure of recurrent laryngeal nerve, and careful dissection reduce the risk of such permanent injury. Hypoparathyroidism is also considered as one of the mostly commonly seen complications related to thyroid surgery. In subtotal thyroidectomy, the surgeon does not approach near the recurrent laryngeal nerve but in total thyroidectomy, the entire tissue of the thyroid gland is removed. For the same reason, the risk of hypoparathyroidism is increased in total thyroidectomy because the parathyroid glands are at risk due to the complete removal of the gland. Thus, surgical procedures like total thyroidectomy which involves high risk require special skills, surgical training, and proficiency to reduce complications associated with it.

In a study done by Pradeep et al., incidences of complications of thyroidectomy were temporary hypocalcemia (24%), permanent hypocalcemia (3%), and permanent vocal cord palsy (1%) [19]. When parathyroids are incidentally removed, or the tumor invades the capsule and end artery of parathyroid, hypocalcaemia is expected to be seen [21, 22].

The prevalence of postoperative hypocalcemia following thyroidectomy which may be temporary or permanent ranges from 0% to 83%, with the highest incidence seen in patients undergoing total thyroidectomy for cancer (28%) and in those who underwent subtotal thyroidectomy for thyrotoxicosis (23%). On the contrary, the incidence of hypocalcemia is found to be lowest in patients undergoing subtotal thyroidectomy for other diseases (1.5%) and lobectomy (0%) [23].

The percentage of total thyroidectomies being performed for various thyroid diseases has increased significantly in recent years. Initially, the risks which were associated with major surgeries to treat thyroid disease prevented the surgeons from performing total thyroidectomies. Although the use or risks associated with total thyroidectomy remain controversial it is being performed increasingly [24]. Kaniuka et al. have described how iodine treatment in multinodular goiter might be a good alternative to surgery [25]. In older patients, subtotal thyroidectomy may be the best optional procedure so as to avoid their total and permanent dependence on drugs [26]. Some authors favor the subtotal procedure because of its lower incidence of iatrogenic nerve injuries [27]. Efremidou et al. opine that total thyroidectomy is a safe and valued surgical procedure for the management of several benign thyroid diseases, particularly in patients who are at increased risk of recurrence and they believe that hemostasis occurs better after total thyroidectomy [28]. Bage et al. believe that total thyroidectomy is a safe operation for treating bilateral benign multinodular goiter as it provides a definitive cure of the disease, without any risk of recurrence, and also promises of total relief of any compressive symptoms associated with it and comparably low occurrence of major complications [29].

New techniques such as postoperative levothyroxine therapy seem to reduce recurrence rates in the other thyroid lobe after partial resection of the thyroid gland [30]. But not all new techniques have shown promising results. For example, the use of an ultrasonic dissector does not seem to influence complication rates, as reported in two studies [31, 32].

In the present phase, thyroid gland diseases are one of the most encountered endocrine disorders especially in south Indian population. This factor may be due to the decreased iodine intake of that region or differences in geographical distribution or any other factors. Mathew believes that there is a high burden of thyroid diseases in India but paucity of data is available on the epidemiology of thyroid diseases [33]. He opines that, due to lack of resources, screening for thyroid diseases in the general population is not cost effective. Because of the high cost and limited availability of radioactive iodine, thyroidectomies are preferred and common in India. Hence in our review we found that total thyroidectomy is safe and cost effective with low complication rates which should encourage this modality of treatment in Indian population. New advanced techniques like endoscopy are also available now for early diagnosis as well as for thyroid surgery. Few minimally invasive approaches to thyroidectomy and also advanced procedures like use of surgical robots have also been tried. But there exists a controversy over the role of the robot in thyroidectomy because traditional thyroidectomy procedure has shown to have a low morbidity rate and produces excellent results. Improved cosmetics are the main advantage of robotic thyroidectomy. Limitations of robotic thyroidectomy include longer operative time, increased equipment and staffing needs, and complications related to the surgical approach, such as injury to brachial plexus [34].

Complications after thyroidectomy are in a decreasing trend and most of the complications could be avoided by careful dissection and exposure of anatomical structures and proper surgical management.

## 5. Conclusion

Based on the above evidences, we would conclude from our review that total thyroidectomy is a safe and effective procedure for most of the thyroid diseases in the hands of an expert surgeon. Subtotal thyroidectomy is similarly effective but it is associated with significant recurrence rate and may leave few traces of inadequately treated thyroid cancers. Thus total thyroidectomy provides little significant advantage of being safer procedure compared to subtotal thyroidectomy.

## Figures and Tables

**Table 1:** Incidence of recurrent laryngeal nerve injury in various thyroid procedures.

Study	Surgical procedure	Incidence of permanent recurrent laryngeal nerve injury
Barczyński et al. [35]	TT versus BST	5.49% versus 2.1%

Vaiman et al. [36]	TT versus NTT TT versus STT	1.4% versus 1.2% 1.4% versus 1.1%

Tezelman et al. [37]	TT versus BST	No significant difference

Vaiman et al. [38]	TT versus STT TT versus reoperation	1.4% versus 1.2% 1.4% versus 3%

Yang et al. [39]	TT versus STT	1.89% versus 1.68%

Zaraca et al. [40]	TT versus NTT	Not reported

Ozbas et al. [41]	TT versus NTT	1.9% versus 0.6%

Erbil et al. [42]	TT versus NTT	0.9% versus 0.9%

TT: total thyroidectomy; STT: subtotal thyroidectomy; NTT: near total thyroidectomy; BST: bilateral subtotal thyroidectomy.

## References

[1] Standring S. (2008). *Gray's Anatomy: The Anatomical Basis of Clinical Practice*.

[2] Kochupillai N. (2000). Clinical endocrinology in India. *Current Science*.

[3] Bellantone R., Lombardi C. P., Bossola M. (2002). Total thyroidectomy for management of benign thyroid disease: review of 526 cases. *World Journal of Surgery*.

[4] Bron L. P., O'Brien C. J. (2004). Total thyroidectomy for clinically benign disease of the thyroid gland. *British Journal of Surgery*.

[5] Loch-Wilkinson T. J., Stalberg P. L. H., Sidhu S. B., Sywak M. S., Wilkinson J. F., Delbridge L. W. (2007). Nerve stimulation in thyroid surgery: is it really useful?. *ANZ Journal of Surgery*.

[6] Gough I. R., Wilkinson D. (2000). Total thyroidectomy for management of thyroid disease. *World Journal of Surgery*.

[7] Jessie W. U., Harrison B. (2010). Hypocalcemia after thyroidectomy: the need for improved definitions. *World Journal of Endocrine Surgery*.

[8] Perzik S. L. (1976). The place of total thyroidectomy in the management of 909 patients with thyroid disease. *The American Journal of Surgery*.

[9] Gough I. R. (1992). Total thyroidectomy: indications, technique and training. *Australian and New Zealand Journal of Surgery*.

[10] Delbridge L., Guinea A. I., Reeve T. S. (1999). Total thyroidectomy for bilateral benign multinodular goiter: effect of changing practice. *Archives of Surgery*.

[11] van Zuidewijn D. B. W. D. R., Songun I., Kievit J., van de Velde C. J. H. (1995). Complications of thyroid surgery. *Annals of Surgical Oncology*.

[12] Liu Q., Djuricin G., Prinz R. A. (1998). Total thyroidectomy for benign thyroid disease. *Surgery*.

[13] Cirocchi R., Trastulli S., Randolph J. (2015). Total or near-total thyroidectomy versus subtotal thyroidectomy for multinodular non-toxic goitre in adults. *Cochrane Database of Systematic Reviews*.

[14] Beahrs O. H., Vandertoll D. J. (1963). Complications of secondary thyroidectomy. *Surgery, Gynecology & Obstetrics*.

[15] Tsegaye B., Ergete W. (2003). Histopathologic pattern of thyroid disease. *East African Medical Journal*.

[16] Der E. M., Quayson E., Clegg-Lamptey J. N., Wiredu E. K., Ephraim R. K. D., Gyasi R. K. (2013). Thyroid disorders in Accra, Ghana: a retrospective histopathological study at the Korle-Bu teaching hospital. *Journal of Medical and Biomedical Sciences*.

[17] Rumstadt B., Klein B., Kirr H., Kaltenbach N., Homenu W., Schilling D. (2008). Thyroid surgery in Burkina Faso, west Africa: experience from a surgical help program. *World Journal of Surgery*.

[18] Feehery J. M., Pribitkin E. A., Heffelfinger R. N. (2003). The evolving etiology of bilateral vocal fold immobility. *Journal of Voice*.

[19] Pradeep P. V., Agarwal A., Baxi M., Agarwal G., Gupta S. K., Mishra S. K. (2007). Safety and efficacy of surgical management of hyperthyroidism: 15-year experience from a tertiary care center in a developing country. *World Journal of Surgery*.

[20] Katz A. D., Bronson D. (1978). Total thyroidectomy: the indications and results of 630 cases. *The American Journal of Surgery*.

[21] Reeve T. S., Delbridge L., Brady P., Crummer P., Smyth C. (1988). Secondary thyroidectomy: a twenty-year experience. *World Journal of Surgery*.

[22] Haugen B. R., Alexander E. K., Bible K. C. (2016). 2015 American Thyroid Association management guidelines for adult patients with thyroid nodules and differentiated thyroid cancer: the American Thyroid Association guidelines task force on thyroid nodules and differentiated thyroid cancer. *Thyroid*.

[23] Thomusch O., Machens A., Sekulla C., Ukkat J., Brauckhoff M., Dralle H. (2003). The impact of surgical technique on postoperative hypoparathyroidism in bilateral thyroid surgery: a multivariate analysis of 5846 consecutive patients. *Surgery*.

[24] Burnett H. F., Mabry C. D., Westbrook K. C. (1977). Hypocalcemia after thyroidectomy: mechanisms and management. *Southern Medical Journal*.

[25] Kaniuka S., Lass P., Sworczak K. (2009). Radioiodine—an attractive alternative to surgery in large non-toxic multinodular goitres. *Nuclear Medicine Review*.

[26] Pelizzo M. R., Bernante P., Toniato A., Fassina A. (1997). Frequency of thyroid carcinoma in a recent series of 539 consecutive thyroidectomies for multinodular goiter. *Tumori*.

[27] Foster R. S. (1978). Morbidity and mortality after thyroidectomy. *Surgery Gynecology and Obstetrics*.

[28] Efremidou E. I., Papageorgiou M. S., Liratzopoulos N., Manolas K. J. (2009). The efficacy and safety of total thyroidectomy in the management of benign thyroid disease: A review of 932 cases. *Canadian Journal of Surgery*.

[29] Bage A., Bage N., Anand K., Vijayasundaram (2012). Total thyroidectomy versus subtotal thyroidectomy in multinodular goitre—our experience. *The Internet Journal of Otorhinolaryngology*.

[30] Barczyński M., Konturek A., Golkowski F., Hubalewska-Dydejczyk A., Cichoń S., Nowak W. (2010). Five-year follow-up of a randomized clinical trial of unilateral thyroid lobectomy with or without postoperative levothyroxine treatment. *World Journal of Surgery*.

[31] Leonard D. S., Timon C. (2008). Prospective trial of the ultrasonic dissector in thyroid surgery. *Head and Neck*.

[32] D'Ajello F., Cirocchi R., Docimo G. (2010). Thyroidectomy with ultrasonic dissector: a multicentric experience. *Il Giornale di Chirurgia*.

[33] Mathew J. (2008). Burden of thyroid diseases in India. Need for aggressive diagnosis. *Medicine Update*.

[34] Sun G. H., Peress L., Pynnonen M. A. (2014). Systematic review and meta-analysis of robotic vs conventional thyroidectomy approaches for thyroid disease. *Otolaryngology—Head and Neck Surgery*.

[35] Barczyński M., Konturek A., Hubalewska-Dydejczyk A., Golkowski F., Cichoń S., Nowak W. (2010). Five-year follow-up of a randomized clinical trial of total thyroidectomy versus Dunhill operation versus bilateral subtotal thyroidectomy for multinodular nontoxic goiter. *World Journal of Surgery*.

[36] Vaiman M., Nagibin A., Hagag P., Buyankin A., Olevson J., Shlamkovich N. (2008). Subtotal and near total versus total thyroidectomy for the management of multinodular goiter. *World Journal of Surgery*.

[37] Tezelman S., Borucu I., Senyurek Y., Tunca F., Terzioglu T. (2009). The change in surgical practice from subtotal to near-total or total thyroidectomy in the treatment of patients with benign multinodular goiter. *World Journal of Surgery*.

[38] Vaiman M., Nagibin A., Olevson J. (2010). Complications in primary and completed thyroidectomy. *Surgery Today*.

[39] Yang W., Shao T., Ding J. (2009). The feasibility of total or near-total bilateral thyroidectomy for the treatment of bilateral multinodular goiter. *Journal of Investigative Surgery*.

[40] Zaraca F., Di Paola M., Gossetti F. (2000). Benign thyroid disease: 20-year experience in surgical therapy. *Chirurgia Italiana*.

[41] Ozbas S., Kocak S., Aydintug S., Cakmak A., Demirkiran M. A., Wishart G. C. (2005). Comparison of the complications of subtotal, near total and total thyroidectomy in the surgical management of multinodular goitre. *Endocrine Journal*.

[42] Erbil Y., Barbaros U., Salmaslıoğlu A., Yanik B. T., Bozbora A., Özarmağan S. (2006). The advantage of near-total thyroidectomy to avoid postoperative hypoparathyroidism in benign multinodular goiter. *Langenbeck's Archives of Surgery*.

